# *Advances in nanotechnology and the benefits of using cellulose* nanofibers in animal nutrition

**DOI:** 10.14202/vetworld.2021.2843-2850

**Published:** 2021-11-03

**Authors:** Geovane Rosa de Oliveira, Carla de Andrade, Cristina Santos Sotomaior, Leandro Batista Costa

**Affiliations:** Graduate Program in Animal Science, School of Medicine and Life Sciences, Pontifícia Universidade Católica do Paraná, PUCPR, Curitiba - PR, Brazil

**Keywords:** animal performance, animal production, coproduct, fibers, nanocellulose

## Abstract

The production of cellulose nanofibers promotes the utilization of plant residues that are generated in agro-industries during food processing. The utilization of these plant by-products reduces environmental contamination. Cellulose nanofibers are used in several sectors, including the drug, food, and animal nutrition industries. Many sources of nanofibers used in animal diets can be used as potential fiber substitutes after being processed to improve efficiency. For instance, including nanometric particles of plant fibers (<100 nm) in animal feed may provide excellent physical properties such as high reactivity, a large surface area, and improved nutrient absorption from the diet. Nanotechnology improves the characteristics of fibers that are important for gastrointestinal transit and their utilization as energy sources and substrates for microbial fermentation in the digestive tract of animals. Nanofibers can improve the synthesis of volatile fatty acids and the blood lipid profile, with positive effects on the intestinal health of animals. Moreover, *in vitro* and *in vivo* studies have demonstrated promising effects in reducing blood glucose levels without toxic effects on the body. Supplying nanofibers in the diet improve animal performance, increase productivity, and work toward a more sustainable economic development of agribusinesses. The quality of animal products such as meat, milk, and eggs is also reported to be improved with the inclusion of nanominerals in the feed. Overall, the application of nanotechnology to harness the by-products of agro-industries can increase economic viability and sustainability in animal production systems. Therefore, this review presents a current survey on the main research and advances in the utilization of nanotechnology, focusing on cellulose nanofibers in animal feed to improve animal performance.

## Introduction

Many currently available products contain materials in nanometric dimensions, such as carbon nanotubes or synthetic amorphous silica, and several others, such as cellulose nanomaterials are being developed. Cellulose is the most abundant semi-crystalline polymer in nature; it covers a wide spectrum of structures with different shapes, sizes, and chemical surfaces [1]. Nanofibers (NFs) are stable nanometric structures corresponding to 1 billionth of a meter (1 nm=10^–9 m^) with dimensions less than 100 nm, yet greater in length. Nanofibers can be obtained from various cellulosic plant sources by mechanical, chemical, or enzymatic methods [2,3], for producing materials with increased surface areas, reactivity [4], and absorption. Cellulose nanofibers have several ­applications in the food, packaging, and biomedicine industries. In addition to being obtained from biodegradable natural sources, their production and processing is low cost. The global nanocellulose (NC) market is estimated to grow by more than 18% by 2023, reaching a mark of 661.3 million dollars [5]. One of the key factors driving the global development of cellulose nanostructures is the growing demand for sustainable products. These aspects have encouraged new studies to understand the reactivity of cellulose nanofibers with proteins, fats, enzymes, as well as animal cells and tissues [6,7]. Overall, nanotechnology improves the use of plant by-products in a sustainable manner by increasing the dietary fiber utilization capacity, thus benefiting animal production.

Nanofibers have been reported to exert healing effects [8,9], bone regeneration [10,11], and anti-inflammatory effects on intestinal epithelial cells, indicating their biomedical applications [12]. Further, they show increased absorption in enterocytes, mediated by specific transporters [13,14]. However, the application of nanotechnology in animal nutrition is relatively recent, with few studies on the potential effects of nanoparticles, and their ability to react with bacteria in the digestive tract, based on their catalytic, magnetic properties, and reactivity [15]. In the previous studies [6,16,17], nanofibers from pupunha palm heart were found to increase the body weight of rats by 9%, without any toxic effects to the animals [16], no histopathological changes, and no effect on feed consumption [17]. It was observed that serum triglyceride (TG) values were reduced by 36.0% when 1.0% NC was included in the diet of rats [6]. Cellulose nanofibers reduce the contact for the action of lipases, decreasing the digestion and absorption of fats in the small intestine, thereby improving the blood lipid profile. Nanominerals are widely used in diversified sectors, including agriculture, animal, and food systems. These nanominerals have significant growth-promoting, immunomodulatory, antibacterial effects even at much lower doses than the conventional organic and inorganic mineral sources [18,19]. In poultry diets, supplementation with nanominerals (selenium, zinc, and chromium) promotes weight gain, egg production, and improved product quality [18]. Nano-selenium and nano-zinc in the diet of laying hens improved egg production [20] and bone strength [21]. In addition, increased body weight and feed efficiency have been reported with the inclusion of nano-chrome in the diet of birds [22]. In broilers, nano-selenium is reported to improve weight gain [23] and meat quality [24], in addition to reducing the negative effects of heat stress [25]. In sheep, supplementation with nano-selenium improved ruminal fermentation and volatile fatty acids (VFA) synthesis [26], thus increasing microbial activity and the use of nutrients from the diet [27]. Supplementation of chitosan nanoparticles in the diet of piglets improved the average daily weight gain of the animals by 38.31 g [28]. In an *in vitro* study on esophageal cells from rats [29], it was observed that the adsorbent effect of NC reached up to 85% for aflatoxin B_1_ in different foodstuffs (hazelnut, pistachio, almond, walnut, wheat, and rice), without any toxic effect on the esophagus. Considering the health benefits that can be achieved with nanofibers supplied from abundant and renewable natural sources, the use of agro-industrial waste contributes to sustainability in animal production and nutrition systems with increased productivity and reduced environmental impact.

Within this context, the use of nanotechnology to obtain nanofibers with different functional properties is promising, as they present increased reactivity and can interact with the intestinal epithelium layer, thus improving the use of fibers, which are mostly indigestible by monogastric animals. Nanofibers can help protect the mucosa and improve intestinal health and microbial fermentation in the large intestine. Therefore, production-processing safety in relation to nanofibers must be understood for contributing to food production worldwide. Nanotechnology can thus be used to improve the nutritional aspect of fibers and positively impact the intestinal health of animals.

## Application of Cellulose Nanofibers in Animal Nutrition

Fiber intake is well known to benefit animal health through improved blood lipid profiles, improved intestinal health, and efficient use of the diet [14,30-32], resulting in enhanced performance and productivity rates. Considering the benefits of dietary fiber, several studies have demonstrated the effects of using cellulose nanofibers as an alternative ingredient to improve diet quality and acceptability and promote intestinal health.

Cellulose nanofibers are a new material with excellent potential for use in diets because of their reactive properties and resistance to gastric digestion [33,34], which may favor the integrity of the intestinal mucosa, modulate the digestive tract microbiota, and ensure a healthy balance in the body. Recently, nanofibers were tested as a potential food ingredient for rats, and they were found to maintain adequate growth. Growing rats fed with 7%, 14%, and 21% of nanofibers from the pupunha palm heart sheath showed good acceptability to the feed, without displaying any signs of toxicity, such as vomiting and/or diarrhea [16]. In another study on rats, using 21% of pupunha NC as a potential food ingredient, the performance and structural histology of the liver, glycemia, cholesterol, and TG values remained unchanged [17].

Another effect related to plant-based nanostructures (nano-celluloses) was the mycotoxin adsorbent potential (aflatoxin B_1_) and reduced contamination in rat esophageal cells *in vitro*. Adsorption of aflatoxin B_1_, attributed to its reactive capacity, has also been confirmed in different foodstuffs such as hazelnuts, pistachios, nuts, and rice [28]. In addition, an *in vitro* study indicated that NC concentrations above 1000 mg/mL can induce metabolic disturbance in the cell wall and cell membrane of bacterial and fungal strains by forming a protective barrier in the bacterial cell wall and reducing the metabolism and exchange of cytoplasmic material [35]. It is hypothesized that NC does not cause disturbances or changes in the bacterial cytoplasm but can inhibit the growth of bacteria and fungi, including *Escherichia coli, Streptococcus aureus*, and *Aspergillus niger*.

Intake of cellulose nanofibers can prevent excess fat accumulation in the blood; for instance, an *in vivo* study in rats fed with a diet including 1.0% of NC, showed that the serum TGs were reduced by 36.0%. In an *in vitro* study, a 48.4% reduction was observed in the digestion and absorption of free fatty acids and TGs in the small intestine of an acellular simulated gastrointestinal tract (GIT) [6], thereby avoiding excess body fat. This favors the improvement of blood lipid profiles, with an important contribution to individual health, similar to the use of fibers in the diet [30]. These studies suggest that nanofibers react with digestive enzymes, proteins, and dietary fats during digestion, an important feature to improve the applicability of plant-based nanostructures in animal diets.

## Digestion, Absorption, and Metabolism of Cellulose Nanofibers in the GIT

After ingestion, nanofibers are exposed to various pH conditions and chemical reactions during the digestive process in the mouth, stomach, small intestine, and large intestine. Cellulose nanofibers show chemical differences in their structures, which may be related to the surface area per unit mass or specific surface area [6], with increased absorption during passage through the digestive tract.

The ingested nanoparticles, due to their physicochemical properties and structural characteristics, act in different ways in the GIT of animals. Cellulose nanofibers can influence: (i) Interactions between the components of gastrointestinal fluids (such as enzymes, bile salts, phospholipids, and biopolymers); (ii) the formation of protein and fat aggregates and stability within different regions of the GIT; (iii) the passage of compounds through the mucus layer that lines the intestinal wall; (iv) transport into the cells through the epithelium; and (v) interactions between the fermenting bacteria of the cecum and colon in the large intestine [36-38].

In the mouth, nanoparticles are exposed to mastication whereby positively charged particles interact with saliva proteins [38]. In the stomach, absorption and hydrolysis by digestive enzymes are unlikely due to the thick mucus layer and pH of the environment [33], which prevents the passage of nanoparticles into the bloodstream. Cellulose nanocrystals (CNCs) can reduce the kinetics or initial enzymatic reaction time of proteolytic enzymes to digest proteins in the gastric phase [39].

During passage through the small intestine, nanofibers can penetrate the mucus layer [34] and become desulfated [7]. Furthermore, negatively charged particles can aggregate with other structures [40], facilitating permeability through the digestive tube. In the large intestine, nanoparticles are suggested to interact with the microbiota present, altering the synthesis and metabolism of VFA [41].

The formation of nanofiber aggregates with proteins and fats can alter the action of proteases and lipases in the digestive process [6]. It has been reported that CNCs can form a coating around fat droplets, reducing the solubilization of fats by bile salts (necessary for fixing lipase in digestion). This results in a decreased available surface area for lipase binding and action [39,42], thus reducing the absorption of lipids [3].

The absorption of nanoparticles in the GIT depends on their diffusion through the mucosal lining, initial contact with the intestinal epithelium, and the process of capture by enzymes and translocation through the cell [43]. However, smaller anionic particles with negative polarity may tend to diffuse through the mucus layer and reach the epithelial surface more easily than larger particles [34,44]. The mucus layer throughout the GIT is negatively charged [45], as is sulfated CNCs [40], such as those isolated by chemical processes (H_2_SO_4_). Therefore, the mucus layer and digestive tract epithelium are the main barriers to the passage of nanostructures into the bloodstream [44].

**Figure-1:**
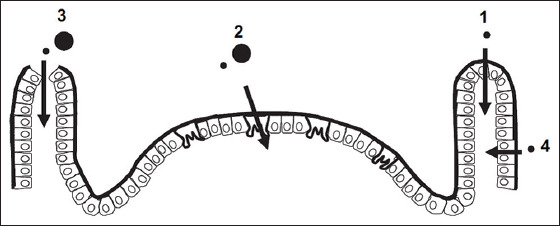
Transcellular route, at the apex of the intestinal epithelium (pinocytosis): (1) Endocytosis. (2) M-cell uptake (transcytosis). (3) Persorption. (4) Putative paracellular uptake [52].

Permeability of the tight junctions in the gastrointestinal epithelium can be modulated by specific polymers and simple organic molecules. These polymers can act as tight junction regulators, thus introducing a gateway for many particles such as toxins, metabolites, and nanoparticles [46]. Another possible uptake route is the transcellular route, at the apex of the intestinal epithelium (pinocytosis), transported through the M cells in the enterocytes (Figure-1), and later released on the basolateral side of the intestinal epithelium [47-49]. In addition, M cells can absorb particles smaller than 200 nm and can thus absorb nanoparticles, which are smaller in size [50,51]. However, absorption has widely been described in quantitative terms in a previous study [52].

After passing through the intestinal epithelium, CNCs can bind various blood components (plasma proteins, clotting factors, platelets, and red and white blood cells) depending on their chemical structure, resulting in the translocation of the particles into organs such as the liver, kidneys, and spleen [53-55]. However, the vast majority of CNCs considered to be absorbed by one of the endocytosis processes [34], are transported by the lymphatic system because of the size of these particles, which can pass through the pores of the vascular system [56].

In addition, Koshani and Madadlou [34], reported that it is unlikely that CNCs participate in the primary and secondary metabolic reactions in the liver due to their considerable hydrophilic nature. However, it has been reported that polystyrene nanofibers, administered intravenously to rats, are absorbed by the liver and excreted in the bile, and that smaller particles (50 nm) are phagocytosed and absorbed by hepatocytes [57]. In another study, systemic distribution of 50 nm and 100 nm nanoparticles in the body, liver, spleen, and blood were reported [58]. Thus, the capture and distribution of nanoparticles in the body depend on the surface characteristics and size of the particles [47]. The structure of the CNCs carried through the organism is practically maintained. This peculiarity of nanofibers can assist several reaction processes in the organism because of their reactive capacity [33], differentiating them from any other ingredient used to improve nutrition at present.

## Toxicological Studies with Cellulose Nanofibers

Understanding the potential implications of nanomaterials, whether beneficial or toxicological, is of paramount importance for developing new technologies in animal and human nutrition. No evidence of NC toxicity has been found in several models of cell and animal exposure in recent studies [59,60]. In *in vivo* studies, inclusion of up to 21% of nanofibers from the pupunha palm heart sheath did not cause histopathological changes in the liver, diarrhea, or vomiting [16,17]. Likewise, supplementation with 100 mg/mL of cotton cellulose nanofibers showed no cytotoxic effect. However, concentrations of 200, 400, 800, and 1000 mg/mL caused cell death in bovine fibroblasts, and high concentrations of CNF (2000 and 5000 mg/mL) decreased cell viability as well as the expression of molecular markers associated with apoptosis [61]. The cytotoxicity of CNCs is less intense than that exerted by structures such as multi-walled carbon nanotubes and crocidolite asbestos [62]. This makes nanofibers a safer ingredient for innovative technologies in animal nutrition.

Using a THP-1 cell line differentiated from human macrophages to screen for cytotoxicity, reported that unlike carbon nanotubes and zinc oxide, cellulose nanofibers were not cytotoxic (at doses up to 100 mg/mL) [63]. In another model of triple cell culture of the human epithelial airway, the cotton cellulose nanofibers showed less potential for cytotoxicity in the synthesis of pro-inflammatory responses compared to multi-walled nanotubes and crocidolite asbestos fibers [62]. Moreover, exposure to cotton NFs (50 and 100 mg/mL) affected the viability and growth of *Chlorella vulgaris* algae after 24 h of exposure. Cytotoxic effects can cause cell death by inducing oxidation, altering cell balance, adenosine triphosphate synthesis, and decreasing photosynthetic activity [63], due to gel formation at high concentrations that block gas exchange across the cell membrane.

The distribution of nanoparticles in the bloodstream can induce an increase in the non-specific immune response. The effects of chitosan nanoparticles were evaluated at doses of 100, 200, and 400 mg/kg on the humoral immune response of growing piglets, and it was observed that the plasma concentrations of IgA and IgG immunoglobulin were higher in animals treated with 400 mg of chitosan nanoparticles [28]. Other characteristics such as particle size and distribution, particle load, and surface area should also be considered when describing the dosage and degree of toxicity of these materials [43]. Considering these results, replacing dietary fiber with cellulose nanofibers may improve the immune status, intestinal microbiota, and performance of animals.

## Innovations and Benefits of Using Nanoparticles in Animal Nutrition

Despite being relatively new to the field, studies have shown beneficial effects of the addition of nanofibers in animal diets (Table-1), highlighting the advantages and benefits of their application [6,17,63], which makes this theme innovative and extremely important for sustainable animal production in an increasingly competitive market.

**Table-1:** Inclusion of nanoparticles in the animal diet as ingredients, and the impacts on animal production.

Reference	Year	Types of nanostructures	Size (nm)	Dose	Animal species	Performance and productivity	Other benefit	Quality of the product	Conclusion
[26]	2011	Nano-selenium	80	3.00 g	Sheep	<pH ruminal<ammonia concentration/N		N/A	>Use of feed nutrients
[27]	2012	Nano-selenium	80	4.00 g	Sheep	<pH ruminal<ammonia concentration	Increased AGVs	N/A	>Digestibility of MS>Feed efficiency
[23]	2012	Nano-selenium	80	0.03 g	Broilers	>Meat quality	Antioxidant effect	N/A	>1.0 mg can cause performance losses
[16]	2013	Pupunha nanofibers	<100	14%	Rats	9% increase in body mass	No toxic effects Good diet acceptability	N/A	>Body weight, homeostatic body balance
[22]	2013	Nano-chromium	80	0.0005 g	Laying chicken	>Body weight, feed intake, and feed efficiency	>Egg production	>Egg quality	>Laying productivity
[20]	2015	Nano-selenium	<80	0.00025 g	Laying chicken	>Egg production>Food conversion	>Antioxidant enzymes activity	Improved egg quality	>Performance and productivity
[17]	2015	Pupunha nano-cellulose	<100	14%	Rats	10% increase in body mass	<Diet consumption; no toxic effects	N/A	>Body weight, without damage to health
[25]	2016	Nano-selenium	80	0.003 g	Broilers	N/O	< Thermal stress effect	N/A	Assists in body balance
[24]	2018	Nano-selenium	80	0.003 g	Broilers	>Productivity indexes>Food conversion	>Weight of breast and drumstick	>Carcass quality	>Chicken development
[21]	2018	Nano-zinc	<80	0.080 g	Laying chicken	< Feed consumption>Egg mass>Shell strength	>Bone resistance	>Egg quality	>Productive performance of layers
[28]	2020	Chitosan nanoparticles	50	0.04 g	Piglets	Increase of 38.82 g GMD weight	Increase of IgA and IgG	N/A	>Performance and the piglet immune system

The studies are structured in chronological order. The table shows the main results related to the parameters studied, including improved performance, conversion and feed efficiency, cytotoxicity, oxidative stress, and improved quality of products of animal origin. The signs: <is equal to lesser or worse; >is equal to greater or better; N/A=Not analyzed, N/O=Not observed. The final column highlights a succinct consideration of the results of the referenced studies

In animal nutrition, application of cellulose nanofibers represents a major advantage. Cellulose nanofibers have a strong ability to penetrate intestinal epithelial cells, due to their size and total surface area, with anti-inflammatory effects, as reported previously [13,14,43,64]. In addition, nanofibers present increased absorption in enterocytes, mediated by specific transporters. It has thus been increasingly accepted that nanofibers can help improve intestinal morphology, as they prevent shortening of villi and tissue injuries, hence improving animal performance.

Satisfactory results were obtained by Andrade *et al*. [17] who observed a 10% increase in the body weight of rats, with no changes in feed intake or damage to the animals’ organs. Mendes [16] observed good acceptability of the diet and increased body mass in rats by including 14% of nano-pupunha in their feed. In addition, no toxic or harmful effects to the animals were observed. Xu *et al*. [28] found that supplementation with 400 mg/kg of chitosan nanoparticles increased the average daily weight gain of piglets by 38.82 g.

Other studies indicate that nanotechnology applied to improve animal nutrition and productivity has provided great advances. Radwan *et al*. [20] observed that 0.25 ppm of nano-selenium in the diet of laying hens increased egg production and feed conversion. In a study by Abedini *et al*. [21], 80 mg of zinc oxide nanoparticles in the diet of laying hens improved feed consumption, egg mass, and shell resistance. Sirirat *et al*. [22] observed that supplementation with chromium nanoparticles (80 nm) improved egg quality and increased mineral retention (Cr, Cu, Ca, Fe, and P) in the liver of birds.

In a study by Ahmadi *et al*. [24], inclusion of nano-selenium significantly improved weight gain and feed conversion. Furthermore, the use of energy and protein in the diet was more efficient in groups supplemented with 0.3 mg of nano-selenium. Cai *et al*. [23] found that nano-selenium supplementation at a concentration of 0.3 mg in the broiler diet improved meat quality. According to El-Deep *et al*. [25], 0.3 mg of nano-selenium reduced the negative effects of heat stress (weight gain, feed intake, feed conversion ratio, breast muscle weight, and abdominal fat weight) in broilers at elevated temperatures (35±1°C).

Positive effects of nanotechnology to increase dietary efficiency have also been found in studies with other animal species (sheep). Liguang *et al*. [26] reported that the inclusion of nano-selenium (3.0 g) in the diet of male sheep had the ability to regulate rumen pH, decrease the concentration of ammonia (N), and induce increased fiber degradation in the rumen as well as improve the use of protein. Xun *et al*. [27] showed that supplementation of the sheep diet with 4 g of nano-selenium reduced the concentration of ammonia in the rumen and improved the digestion of NDF and protein, contributing to increased production efficiency. Thus, the authors highlighted that nanominerals can be used as the preferred source of minerals in animal nutrition, without weakening animal performance. According to studies performed by our team (unpublished results), replacement of 50% of the dietary fiber by pupunha palm nanofibers improved the performance and intestinal health of rabbits, in addition to decreasing the number of enterobacteria in the cecum of growing rabbits.

There are many positive effects of nanotechnology, in improving animal nutrition by increasing the use of coproducts and the efficiency of dietary ingredients, thus benefiting animal performance. Cellulose nanofibers have chemical characteristics, such as long chains, a large surface area, and hydrogen bonds, which increase their reactivity [7]. These characteristics can modulate fermentation and the microbiota in the digestive tract without any toxicological effects, and still provide benefits to animal performance. However, new research must be conducted to improve the applicability of nanofibers as an ingredient, obtained from cellulose, one of the most abundant sources on the planet.

## Conclusion

The utilization of natural resources in a sustainable manner favors the global economy and reduces the environmental impacts of the agricultural industry, which benefits human health and animal production. Nanotechnology can positively impact the animal production chain, with benefits to the intestinal health of animals, increased productivity, and reduced environmental impacts.

## Authors’ Contributions

CA and LBC: Conception of the review. GRO and CA: Collected literature and wrote the manuscript. CA, CSS, and LBC: Corrected the manuscript. All authors read and approved the final manuscript.
